# Silver(I) 1,10-Phenanthroline Complexes Are Active against *Fonsecaea pedrosoi* Viability and Negatively Modulate Its Potential Virulence Attributes

**DOI:** 10.3390/jof9030356

**Published:** 2023-03-15

**Authors:** Ingrid S. Sousa, Tatiana D. P. Vieira, Rubem F. S. Menna-Barreto, Allan J. Guimarães, Pauraic McCarron, Malachy McCann, Michael Devereux, André L. S. Santos, Lucimar F. Kneipp

**Affiliations:** 1 Laboratório de Taxonomia, Bioquímica e Bioprospecção de Fungos (LTBBF), Instituto Oswaldo Cruz (IOC), Fundação Oswaldo Cruz (FIOCRUZ), Rio de Janeiro 21040-900, Brazil; 2 Laboratório de Biologia Celular, IOC, FIOCRUZ, Rio de Janeiro 21040-360, Brazil; 3 Laboratório de Bioquímica e Imunologia das Micoses, Departamento de Microbiologia e Parasitologia, Instituto Biomédico, Universidade Federal Fluminense, Rio de Janeiro 24210-130, Brazil; 4 Rede Micologia RJ–Fundação de Amparo à Pesquisa do Estado do Rio de Janeiro (FAPERJ), Rio de Janeiro 20020-000, Brazil; 5 Center for Biomimetic and Therapeutic Research, Focas Research Institute, Technological University Dublin, D08 CKP1 Dublin, Ireland; 6 Department of Chemistry, Maynooth University, National University of Ireland, W23 F2H6 Maynooth, Ireland; 7 Laboratório de Estudos Avançados de Microrganismos Emergentes e Resistentes (LEAMER), Instituto de Microbiologia Paulo de Góes, Universidade Federal do Rio de Janeiro (UFRJ), Rio de Janeiro 21941-901, Brazil

**Keywords:** chromoblastomycosis, metal-based drugs, antifungal activity, virulence factors

## Abstract

The genus *Fonsecaea* is one of the etiological agents of chromoblastomycosis (CBM), a chronic subcutaneous disease that is difficult to treat. This work aimed to evaluate the effects of copper(II), manganese(II) and silver(I) complexes coordinated with 1,10-phenanthroline (phen)/1,10-phenanthroline-5,6-dione (phendione) on *Fonsecaea* spp. Our results revealed that most of these complexes were able to inhibit *F. pedrosoi*, *F. monophora* and *F. nubica* conidial viability with minimum inhibitory concentration (MIC) values ranging from 0.6 to 100 µM. The most effective complexes against *F. pedrosoi* planktonic conidial cells, the main etiologic agent of CBM, were [Ag(phen)_2_]ClO_4_ and [Ag_2_(3,6,9-tdda)(phen)_4_].EtOH, (tdda: 3,6,9-trioxaundecanedioate), displaying MIC values equal to 1.2 and 0.6 µM, respectively. These complexes were effective in reducing the viability of *F. pedrosoi* biofilm formation and maturation. Silver(I)-tdda-phen, combined with itraconazole, reduced the viability and extracellular matrix during *F. pedrosoi* biofilm development. Moreover, both silver(I) complexes inhibited either metallo- or aspartic-type peptidase activities of *F. pedrosoi* as well as its conidia into mycelia transformation and melanin production. In addition, the complexes induced the production of intracellular reactive oxygen species in *F. pedrosoi*. Taken together, our data corroborate the antifungal action of metal-phen complexes, showing they represent a therapeutic option for fungal infections, including CBM.

## 1. Introduction

Chromoblastomycosis (CBM), recognized by the World Health Organization (WHO) as a neglected tropical disease, is a chronic and granulomatous mycosis known for producing polymorphic lesions on the skin and subcutaneous tissues [1,2]. The advanced disease stages occasionally lead to amputation of the affected limbs, since complications such as lymphedema, secondary bacterial infections and neoplastic transformation might occur [2,3]. This implantation and ubiquitous mycosis is caused by several dematiaceous fungi, including species of the genus *Fonsecaea*, such as *F. pedrosoi*, the most common CBM agent, as well as *F. monophora* and *F. nubica* [4]. These sibling species generally have differences in the global distribution and ability to produce infections [4,5]. The pathogenicity mechanisms responsible for CBM are barely known. Some potential virulence factors have been described, including thermotolerance, the capability of filamentation and especially the melanin production [2,6,7,8]. In recent years, our group has identified hydrolytic enzymes, such as peptidases, ectophosphatases, phospholipases and esterases, produced by *F. pedrosoi* cells with a multitude of biological roles, including nutrition, proliferation, filamentation and adhesion, which are related to the CBM virulence attributes. Recently, we described the ability of *F. pedrosoi* to form robust biofilm on both abiotic and biotic substrates [9,10,11,12,13,14,15,16,17].

Distinct therapies have been recommended to each CBM stage, including chemotherapy, such as itraconazole and/or terbinafine, surgery, cryotherapy as well as some of their combinations [2,3]. However, no consistent therapy has arisen as a gold standard against CBM due to the high cost, toxicity, long duration and the occurrence of microbial resistance [2]. In this context, the search and design of new antifungal drugs for CBM treatment is a priority. For more than ten years, our research group has studied distinct synthetic bioactive compounds against CBM fungi [11,12,13,15,16,18,19,20,21]. Bearing in mind the drug repositioning therapeutic approaches, we studied several peptidase inhibitors (PIs) and demonstrated that aspartic PIs, used in clinical practice against the human immunodeficiency virus (HIV), jeopardized CBM fungi viability [16,20]. Indeed, the HIV-PIs are rising up as attractive candidates for antifungal therapies [22,23]. Regarding *F. pedrosoi*, our study showed that nelfinavir and saquinavir, in particular, had a great activity against its conidial and sclerotic cells [13,16]. In addition, these HIV-PIs compromised the ultrastructure of *F. pedrosoi* conidia as well as negatively interfered with the aspartic peptidase activity, morphological transition and interaction with host cells [15,16]. Moreover, our group demonstrated that 1,10-phenanthroline (phen), a heterocyclic organic compound and a classical metallopeptidase inhibitor, not only reduced *F. pedrosoi* extracellular zinc metallo-type peptidases, but also hindered its proliferation [11]. In fact, studies have revealed that phen has an antimicrobial broad-spectrum, presenting antiprotozoal, antibacterial and antifungal activities [24,25]. 

Phen is a well-known chelating bidentate ligand used in the coordination chemistry for inorganic drug design [26,27]. Therefore, several studies showed that metal-based complexes containing phen have a remarkable biological action, especially antimicrobial activity [28,29,30]. Regarding the yeasts, metal complexes derived from phen were also able to inhibit the growth of *Candida albicans* and non-*albicans Candida* species, such as *C. glabrata*, *C. tropicalis*, *C. krusei* and *C. haemulonii* [24,31,32,33,34,35,36]. Copper(II) and silver(I) phen complexes of malonic acid reduced the *C. albicans* growth and ergosterol amount as well as damaged its mitochondria [33,37]. In addition, manganese(II), copper(II) and silver(I) phen and their derivatives containing dicarboxylate and perchlorate anions affected the viability of *C. haemulonii* species under both planktonic and biofilm induction conditions [36]. In filamentous fungi, the perchlorate salts of copper(II) and silver(I) complexes of 1,10-phenanthroline-5,6-dione (phendione) inhibited the growth of *Scedosporium apiospermum* (formerly *Pseudallescheria boydii*) [38]. Our study also showed that these complexes were capable of inhibiting the proliferation and filamentation of *Phialophora verrucosa*, another CBM fungus, as well as its viability after in vitro interaction with human macrophages and in vivo infection in *Galleria mellonella* larvae [19,21]. In general, these metal complexes derived from phen/phendione were well tolerated in vitro by mammalian cells, including immune cells like macrophages, as well as in vivo models such as *G. mellonella* larvae and Swiss mice [38,39].

Studies showed that drugs with metal ions in their molecular structures impact medicinal chemistry through their ability to exist in and switch between different oxidation states, achieve geometric structural diversity and the possibility of such metals coordinating to organic drugs, which can significantly improve their actions [5,40,41]. The mechanisms of action of these metal complexes derived from phen/phendione in fungal cells have not been fully elucidated. However, studies have shown that they can disrupt the plasma membrane integrity, damage the mitochondrial function (enzymatic activities and electric membrane potential), cleave nuclear DNA, chelate essential metals, cause rupture of internal organelles and alter the control of cell division [24]. The potential multimodal action of metal chelates differentiates them from classical antifungal agents, which are normally administered to treat CBM, offering an alternative therapy for overcoming the resistance to these antifungal agents. In addition, the possibility of using metal based-drugs combined with classical antifungal agents may improve the activity of the latter and minimize their toxicity, further enhancing the use of such metal-based drugs as a promising therapeutic approach [41,42].

Based on all mentioned premises, in the present work, we aimed to investigate the effects of metal-phen/phendione complexes on the viability of species belonging to *Fonsecaea* genus and their impact on potential fungal virulence attributes of *F. pedrosoi*, such as morphological transition, hydrolytic enzymes, melanin and biofilm, as well as on reactive oxygen species (ROS) production. 

## 2. Materials and Methods

### 2.1. Fungal Growth Conditions 

*Fonsecaea pedrosoi* strain (ATCC 46428, previously known as 5VPL), isolated from a Brazilian patient with chromoblastomycosis, was provided by the Collection of Reference Microorganisms in Sanitary Surveillance (CMRVS) of the National Institute for Quality Control in Health, FIOCRUZ, Rio de Janeiro, Brazil. The clinical isolates of *F. monophora* (CFP 993) and *F. nubica* (CFP 994) were obtained from the Collection of Pathogenic Fungi of Evandro Chagas National Institute of Infectious Diseases, FIOCRUZ. The cultures were maintained at 4 °C on Sabouraud dextrose agar (SDA) medium using mineral oil for preservation. The fungal cells were cultivated in a 100 mL of Czapek-Dox broth medium, pH 5.5 (BD-Difco, Silicon Valley, CA, USA), for 6 days under constant agitation at 26 °C for all assays, except the one for evaluating the metallopeptidase activity. The cultures were centrifuged at 2400× *g* for 10 min, the cells were washed three times in 0.9% NaCl and the number of conidia was determined after counting the cells in a Neubauer chamber [14].

### 2.2. Test Compounds

The metal complexes and the publications with the methodologies employed for their synthesis are detailed in Table 1. In addition, other compounds obtained from Sigma-Aldrich (St. Louis, MO, USA), such as phen, phendione and different salts (silver perchlorate, silver nitrate, copper perchlorate, copper sulfate and manganese chloride), were also tested. All the compounds were solubilized in sodium dimethyl sulfoxide (DMSO, Sigma-Aldrich), except derivatives **12**, **13**, **14** and the salts, which were resuspended in water [34,36].

### 2.3. Antifungal Susceptibility Test

The effect of compounds on the viability of species belonging to *Fonsecaea* genus was assessed using the M38-A2 document for filamentous fungi, as described by the Clinical and Laboratory Standards Institute (CLSI) [49], with some modifications. Briefly, the conidia were added in the 96-well microtiter assay plates with Roswell Park Memorial Institute (RPMI) 1640 medium at pH 7.0 buffered with 0.16 M 3-(*N*-morpholino) propanesulfonic acid (MOPS) containing concentrations ranging from 100 to 0.048 μM of the metal complexes (**1**–**14**). Moreover, complexes **12** and **14** were also tested at concentrations from 10 to 0.0048 μM. After incubation for 72 h at 35 °C, the minimum inhibitory concentration (MIC) was determined by visual inspection and resazurin staining assay [50]. Systems containing fungal cells in RPMI medium and the same medium were solely used as control. Itraconazole was used as the reference antifungal drug at concentrations ranging from 25 to 0.012 μM. As recommended by CLSI, *Candida parapsilosis* (ATCC 22019) were used as quality control. For *F. pedrosoi*, other compounds varying from 100 to 0.048 μM, such as phen, phendione, silver perchlorate, silver nitrate, copper perchlorate, copper sulfate and manganese chloride, were also assessed. To determine the minimum fungicidal concentration (MFC) after defining the MIC, a 2.5 μL aliquot was collected from each well of *F. pedrosoi* plates and transferred to other ones containing SDA medium. MFC was established as the lowest compound concentration without fungal growth. The fungicidal effect was considered when the MFC value ≤ 4 times the MIC value. MFC > 4 times the MIC value means there was a fungistatic effect [51]. The medium RPMI, MOPS, resazurin and itraconazole were obtained from Sigma-Aldrich (St. Louis, MO, USA). 

### 2.4. Effect of Phen and Its Silver Complexes on F. pedrosoi Biofilm

To evaluate the complex action during fungal biofilm development, conidia (1 × 10^6^) were incubated for 72 h at 37 °C in flat-bottom 96-well microplates containing 100 µL of RPMI 1640 medium buffered with MOPS at pH 7.0 in the absence (control) and in the presence of a serial dilution to obtain final concentrations ranging from 2×MIC to 256×MIC of phen and itraconazole, and from ½×MIC to 32×MIC of complexes **12** and **14.** To assess the effect of complexes on mature biofilm disarticulation, conidia were incubated in 96-well microplates containing the same density and medium above. In this case, just after 72 h, when nonadherent cells were removed, the treatment with phen and itraconazole (2×MIC to 256×MIC), as well as the complexes **12** and **14** (2×MIC to 128×MIC) were performed for 48 h. After incubation and washing, the viability of all the systems (treated and untreated cells) was monitored using a colorimetric assay, which measures the metabolic reduction of 2,3-bis-(2-methoxy-4-nitro-5-sulfophenyl)-2H-tetrazolium-5-carboxanilide (XTT; Sigma-Aldrich, St. Louis, MO, USA) [52]. Briefly, the systems were incubated with 0.1 mg/mL XTT and 0.02 mM menadione (Sigma-Aldrich), and after 3 h of incubation at 37 °C in the dark, the formazan dye production was read at 490 nm. 

Biofilm-forming cells and mature biofilm of *F. pedrosoi* were also treated with complexes but using non-inhibitory concentrations, as determined by XTT assay. Thus, fungal biofilm parameters, such as biomass and extracellular matrix, were also evaluated. After treatment and washing to remove nonadherent cells, the systems were fixed with 100 μL of methanol for 15 min and then stained with 100 μL of 0.3% crystal violet solution (Sigma-Aldrich St. Louis, MO, USA) for 20 min. Next, the cells were decolorized with 100 μL of 30% acetic acid for 5 min and then read at 590 nm for biomass quantification [53]. However, extracellular matrix was detected in non-fixed biofilms, when 100 μL of 0.1% safranin (Sigma-Aldrich) in PBS were added to the systems, and incubated for 5 min. After removing the stain excess with 100 μL of 30% of acetic acid, the systems were subjected to reading at 490 nm [17]. Additionally, we evaluated the effect of non-inhibitory concentrations (determined by XTT assay) of itraconazole and the complexes **12** and also **14** combinations on *F. pedrosoi* biofilm viability, biomass and extracellular matrix. All the absorbance values were measured using SpectraMax M3 (Molecular Devices, San Jose, CA, USA). The biofilm MIC (bMIC) was assessed through visual reading, and confirmed when there were 100% of nonviable cells by the XTT assay. 

### 2.5. Effect of Silver Complexes on F. pedrosoi Differentiation 

For inducing the fungal filamentation as described by Granato et al. [19], 1 × 10^6^/mL of conidia were incubated for 48 h at 26 °C in 24-well microplates containing RPMI 1640 medium buffered with MOPS at pH 7.0 in the absence (control) and in the presence of complexes **12** (¼×MIC, ½×MIC and MIC) and **14** (¼×MIC, ½×MIC, MIC and 2×MIC). Non-inhibitory concentrations of the compounds were used and determined by XTT colorimetric assay [52], to guarantee that the effect of the silver complexes on *F. pedrosoi* differentiation was evaluated using viable fungal cells capable of changing their morphology from conidia into filamentous forms. Conidia incubated in RPMI medium at zero-time (non-differentiated cells) were also used as control. Images were acquired using optical microscope Carl Zeiss MicroImaging GmbH.

### 2.6. Effect of Silver Complexes on the Enzymatic Activities of F. pedrosoi

#### 2.6.1. Extracellular Metallo- and Aspartic Peptidase Activities

Conidia were inoculated in 100 mL of Kauffman and Czapek-Dox, both adjusted to pH 5.5, to evaluate the metallo- and aspartic peptidase activities, respectively. The media were incubated at 26 °C for 6 days under constant agitation [11,12]. The cultures were centrifuged at 2400× *g* for 10 min and the supernatants were clarified by filtration on a 0.45 µM membrane. Next, the cell-free supernatants were 100-fold concentrated in an ultrafiltration system (AMICON/Millipore, Burlington, MA, USA) using a 10 kDa exclusion membrane. The experiments were standardized according to the method described by Lowry et al. [54] and for both proteolysis assays, 10 µL of cell-free supernatants equivalent to 10 μg of protein were incubated in the absence (control) or in the presence of 50 μM of complexes **12** and **14**. 

Briefly, for the metallopeptidase assay, all the systems were incubated at 26 °C for 20 h in the microtubes containing 20 mM sodium acetate buffer, pH 5.5, and 0.1% of fluorescein isothiocyanate (FITC)-casein substrate (Sigma-Aldrich) in DMSO. The reaction was interrupted by adding 150 μL of 0.6 M trichloroacetic acid. After 30 min, the supernatant was transferred to a 96-well opaque microplate containing 0.5 M Tris-HCl buffer, pH 8.5, and the spectrofluorometric reading performed [55]. For the aspartic peptidase assay, the systems were added into a 96-well opaque microtiter plate containing buffer (100 mM sodium acetate, pH 4.7, 1 M sodium chloride, 1 mM ethylenediamine tetraacetic acid (EDTA), 1 mM dithiothreitol (DTT), 10% DMSO and 1 mg/mL bovine serum albumin (BSA)) supplemented with 12 µM of the substrate 7-methoxycoumarin-4-acetyl(MCA)-Gly-Lys-Pro-Ile-Leu-Phe-Phe-Arg-Leu-Lys(DNP)-D-Arg amide (cathepsin D fluorogenic substrate, Sigma-Aldrich). After incubation for 30 min at 37 °C, the systems were subjected to reading [56]. Peptidase activities were detected using the spectrofluorimeter FlexStation 3 (Molecular Devices, San Jose, CA, USA) with 485 nm excitation and 535 nm emission for the metallopeptidase, and 328 nm excitation and 393 nm emission for aspartic peptidase. The metallo- and aspartic peptidase activities were calculated based on the standard curve of FITC and MCA fluorophores, respectively. Additionally, reactional systems added with classical inhibitors of metallo- (1mM phen), and aspartic (10 µM pepstatin A) peptidases were also prepared.

#### 2.6.2. Ectophosphatase Activity 

Firstly, conidia (1 × 10^7^) were treated with complexes **12** and **14 **(2×MIC, MIC, ½×MIC and ¼×MIC) and incubated for 20 h in microtubes containing 100 µL of 20 mM sodium acetate buffer, pH 5.5. Sodium orthovanadate (1 mM, Sigma-Aldrich, St. Louis, MO, USA) was used as an acid phosphatase inhibitor. The reaction was started after adding 5 mM of the substrate *p*-nitrophenyl phosphate (*p*-NPP, Sigma-Aldrich). After 1 h at 26 °C, the systems were centrifuged (9500× g) for 1 min and 50 μL of the supernatant from each microtube was transferred to a 96-well microplate containing 50 μL of 2 M NaOH per well. Then, the systems were read at 415 nm and the phosphatase activity was calculated using a standard *p*-nitrophenol (*p*-NP) curve (Sigma-Aldrich). The viability of conidia under the same conditions above was monitored using the XTT assay. Non-treated cells were used as the viability control [10].

#### 2.6.3. Phospholipase and Esterase Activities

Conidia (1 × 10^7^) were incubated for 20 h at 26 °C in 100 µL of RPMI medium in the absence (control) and in the presence of non-cytotoxic concentrations of complexes **12** (¼×MIC, ½×MIC and MIC) and **14** (½×MIC, MIC and 2×MIC), as determined by XTT colorimetric assay [52]. Then, 10 μL of the cell suspension containing 1 × 10^6^ conidia pretreated of each system were transferred to the center of plates containing SDA medium supplemented with 8% egg yolk emulsion (phospholipase activity assay) and peptone agar, pH 6.5, with 0.5% Tween 80 (esterase production assay). In addition, after dripping conidial suspension into the center of both media, the highest concentration of each complex was added and were henceforth known as post-treatment systems. All the plates were monitored daily and the enzymatic activities were determined according to the Pz index, which is the division of the diameter of the colony by the diameter of the colony plus the precipitation zone. Thus, lower Pz values mean higher phospholipase and esterase production [14].

### 2.7. Effect of Silver Complexes on F. pedrosoi Melanin Production

Conidial cells (1 × 10^6^/mL) were incubated in the absence (control) and in the presence of non-inhibitory concentrations (¼×MIC and ⅛×MIC) of the silver(I)-phen complexes and incubated for 5 days at 26 °C. Next, the conidia were fixed with 4% paraformaldehyde buffer for 60 min at 26 °C. After washing with PBS, the cells were blocked for 60 min with 1% BSA in PBS. Then, the systems were additionally incubated for 60 min with the IgM monoclonal antibody to melanin (mAb 6D2, 25 µg/mL in PBS with 1% BSA) [57]. After that, the samples were incubated with anti-mouse IgM-Alexa 488 conjugate (10 μg/mL) for 60 min at 26 °C. After washing, the cells were stained with calcofluor white (Sigma-Aldrich) for 10 min and the systems were visualized using a Zeiss LSM 710, Axio Observer confocal laser microscope (Carl Zeiss Microscopy) 488 nm (Alexa 488) and 405 nm (calcofluor white). Images were assessed using ZEN 2.1 (black) software.

### 2.8. Effect of Silver Complexes on Induction of ROS in F. pedrosoi

Conidia (1 × 10^7^/mL) were incubated for 2 and 24 h at 26 °C in 96-well opaque microtiter plate, containing 100 µL of RPMI medium with non-inhibitory concentrations of the complexes **12** (MIC, ½×MIC and ¼×MIC) and **14** (MIC, 2×MIC and 4×MIC) determined by XTT assay [52]. Then, the systems were centrifuged to remove the medium, and the cells were incubated in a 0.25 M sodium phosphate buffer, pH 7.4, supplemented with 5 μM Amplex Red and 200 μg/mL horseradish peroxidase (Amplex™ Red Hydrogen Peroxide/Peroxidase Assay Kit, Invitrogen, USA). The resorufin fluorescence was monitored at excitation 530 nm and emission 590 nm in a spectrofluorimeter FlexStation 3 (Molecular Devices, San Jose, CA, USA). Antimycin A (Sigma-Aldrich, 2 µM) was used as positive control. A standard curve was performed with hydrogen peroxide and basal fluorescence was subtracted from all the measurements [58]. 

### 2.9. Statistical Analysis

For each evaluation, three independent experiments were performed in triplicates. The data and graphs were analyzed using the program GraphPad Prism version 9.31 and the statistical analysis according to the One-way ANOVA test. Values of *p* ≤ 0.05 were significant.

## 3. Results and Discussion

### 3.1. Effect of Compounds on the Viability of Fonsecaea spp.

All silver(I), copper(II) and manganese(II) complexes (**1**-**14**) coordinated with phen/phendione were able to inhibit the growth of *Fonsecaea* spp., dysplaying MIC values ranging from 0.6 to 100 μM, except for complex **2** that showed MIC values >100 µM for *F. nubica* and *F. pedrosoi* (Table 2). Similarly, previous studies showed that other fungal cells, such as *C. albicans*, *C. haemulonii* and *P. verrucosa*, were sensitive to these metal complexes with phen/phendione ligands [19,33,34,36,43]. 

Although the data have shown that the metal complexes were effective in inhibiting the proliferation of different *Fonsecaea* species, we chose *F. pedrosoi* for further experiments, since it is the main etiologic CBM agent. For *F. pedrosoi*, the growth inhibition tendency among metals had the following order: Ag(I) > Mn(II) > Cu(II). Likewise, Gandra et al. [36] showed that Ag(I) complexes had the best performance at inhibiting the viability of *C. haemulonii* planktonic cells. Thus, our data corroborate that the inhibition of fungal growth can be related to the nature of the metal ion, as previously discussed [34]. The most effective complexes against *F. pedrosoi* were silver(I)-phen (**12**) and silver(I)-tdda-phen (**14**) with MIC values equal to 1.2 µM and 0.6 µM, respectively. It is worth mentioning that, as previously reported, the presence of hydrophilic 3,6,9-tdda in metal-phen complexes enhances their water solubility, and consequently their action against microbial cells [59]. It may, in part, explain the better performance of complex **14** against *F. pedrosoi*. Indeed, this complex was extremely active in inhibiting the growth not only for fungal, but also bacterial and *Mycobacterium tuberculosis* cells [36,59,60,61]. 

Additionally, we showed that both Ag(I)-phen complexes demonstrated a fungicidal effect against *F. pedrosoi* planktonic cells, with MFC values equal to 5 μM for complex **12** and 2.5 μM for complex **14** (Table 2). Our data corroborate the high antimicrobial efficacy of complexes **12** and **14** reported for nine clinical isolates of *C. haemulonii* (geometric mean of MIC values 1.76 and 0.83 µM, respectively) [36]. Previously, we showed that silver complex (**10**) also had antifungal activity against *P. verrucosa*, with MIC equal to 4 µM [19]. However, *F. pedrosoi* was a little less sensitive, showing MIC of 6.2 µM (Table 2). It is well known that phen and phendione are potent chelating ligands, with the distinctive feature of interacting with a variety of transition metal ions, producing thermodynamic stability complexes [26,62]. Previous studies showed that phendione complexes were more bioactive than phen complexes [33,34]. Concerning *C. albicans*, McCann et al. [34] demonstrated that Ag(I) phendione complex (**10**) was more potent (MIC = 0.5 µM) than Ag(I) phen complex (**12**), which had MIC equal to 8.8 µM. Conversely, we showed that the complex of silver(I) with phen (**12**; MIC = 1.2 µM) enhanced anti-*F. pedrosoi* action, more than the one with phendione (**10**; MIC = 6.2 µM) (Table 2). 

To confirm that the anti-*F. pedrosoi* effect observed was due to the metal complexes rather than to the free metal ions, the antifungal activities of the simple metal salts and the metal-free phen and phendione ligands were assessed (Table 3). Previously, our group showed that phen was able to inhibit *F. pedrosoi* growth in concentrations of 0.1, 1 and 10 mM [11,25]. Herein, the phen MIC value (3 μM) for *F. pedrosoi* was determined (Table 3), and this antifungal activity corroborates the data that have already been described for *P. verrucosa* and other fungi [18,25]. Metal-free phen did not have a fungicidal effect displaying MFC >100 µM (Table 3). The results showed that the silver perchlorate and silver nitrate salts were able to inhibit the growth of *F. pedrosoi* with fungicidal effect (Table 3). However, these silver salts showed higher MIC values than silver(I) coordinated to the phen ligand. The complex **12** (MIC = 1.2 μM) was 5 times more effective than the simple silver perchlorate salt (MIC = 6.2 μM). In fact, silver(I)-perchlorate salt complexes, such as **12** and **10**, had a greater anti-*Candida* activity than the free silver ions [34,36]. Furthermore, our data also revealed that the copper perchlorate, copper sulfate and manganese chloride salts did not affect the growth of *F. pedrosoi* (MIC values > 100 μM, Table 3). Instead, these metals had antifungal activity against *F. pedrosoi* when coordinated with phen. These data corroborate previously published results that showed metal complexes had higher antimicrobial activity than free metal ions and metal-free ligands [5,19,36]. 

Considering that complexes **12** and **14** were the most effective fungal growth inhibitors, they were selected to be used in the other experiments herein. It is important to emphasize that, according to Gandra et al. [39], both complexes were well tolerated by *G. mellonella* in concentrations up to 750 mg/L (15 μg/larva). Remarkably, the *F. pedrosoi* MIC values for both silver(I)-phen complexes were much lower, varying between 0.62 and 1.2 µM (for both 0.7 mg/L). This low cytotoxicity also corroborates with the choice of these complexes to further studies. 

### 3.2. Effect of Phen and Its Silver Complexes on the Biofilm Formation and Maturation in F. pedrosoi 

**Figure 1:**
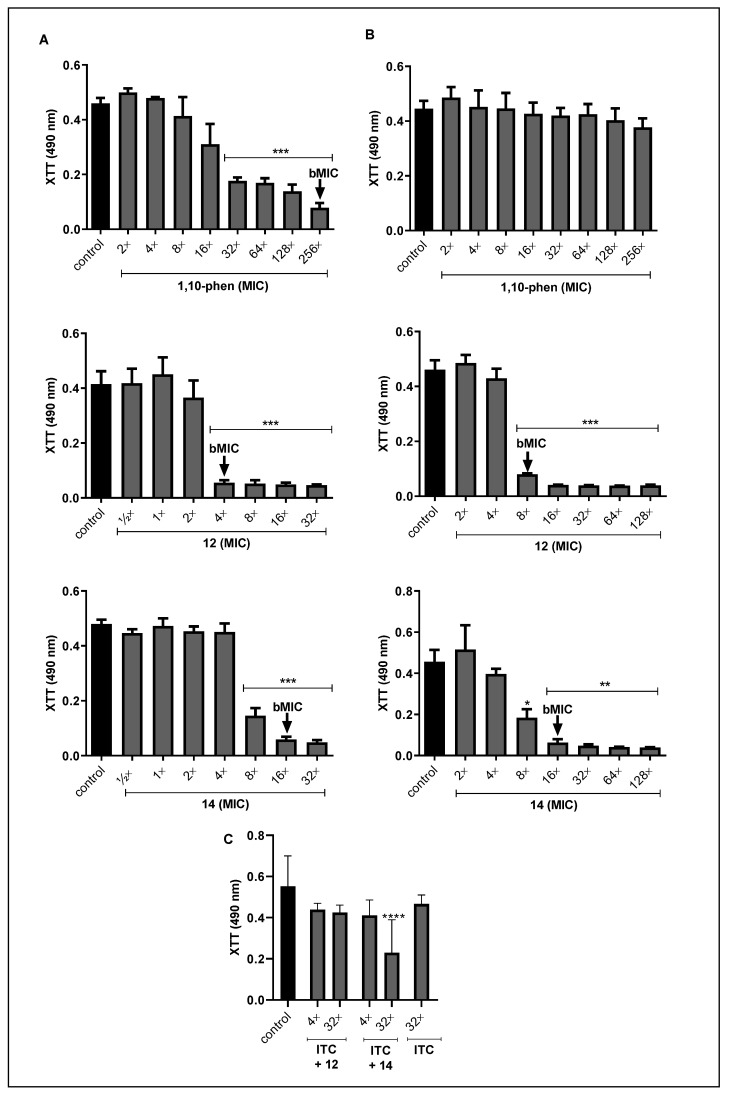
Effect of phen and its silver complexes on the viability of *F. pedrosoi* biofilm formation and maturation. (**A**) In a 96-well polystyrene microplate, conidia were treated immediately with different concentrations of phen and its silver complexes, and incubated in RPMI medium for 72 h at 37 °C; (**B**) Conidia were added in another microplate to form biofilm and, just after 72 h, the test compounds were supplemented and the systems incubated for an extra 48 h; and (**C**) Conidia were incubated for 72 h with a combination of non-inhibitory concentrations of itraconazole and the complexes **12** (2×MIC) and **14** (4×MIC). The cell viability and the minimum inhibitory concentration values of fungal biofilms (bMIC) were assessed using XTT reduction assay after reading at 490 nm [52]. Non-treated conidia were also included as control systems. * *p* < 0.05, ** *p* < 0.01, *** *p* < 0.001, **** *p* < 0.0001.

Recently, we showed that *F. pedrosoi* has the ability to form biofilm on polystyrene substrate, and that 72 h-old biofilm was more resistant to the classical antifungal agents than the planktonic-growing cells [17]. Taking this into account, we evaluated the effects of phen and its silver complexes during the *F. pedrosoi* biofilm formation, and on the disarticulation of mature biofilm. For the first one, phen showed a bMIC value equal to 256×MIC value (794 μM), whereas the complexes **12** and **14** presented 4×MIC value (4.8 μM) and 16×MIC value (9.6 μM), respectively (Figure 1A). We observed that both complexes were more effective at disturbing the fungal viability than the free phen. This better performance of the metal complexes compared to their ligands was also demonstrated against the biofilm formed by *P. verrucosa* and clinical isolates of *C. haemulonii* [19,36]. 

In the mature biofilm condition, phen was not able to affect fungal viability up to the highest concentration tested, presenting bMIC > 256×MIC value, while the silver complexes remained highly active. For complex **12,** the bMIC value was reached when it was 2-fold (9.6 µM) in comparison to that used to inhibit the fungal biofilm during its development. Indeed, *F. pedrosoi* biofilm was more sensitive to complex **12** than the biofilm produced by *C. haemulonii* species, that showed geometric mean (GM) of the bMIC values equal to 21.7 µM [36]. Surprisingly, the same concentration of complex **14** (16×MIC value) was also efficient in inhibiting 100% of *F. pedrosoi* viability under this condition (Figure 1B). Likewise, *C. haemulonii* species were also sensitive to complex **14,** showing GM-bMIC values of 5.2 μM, lower than that found for *F. pedrosoi* [36]. In contrast, at this cellular density (initial inoculum of 10^6^ conidial cells), itraconazole was not able to affect *F. pedrosoi* viability up to 256×MIC value [69 mg/L (100 μM)], even during its biofilm formation (data not shown). These results are in agreement with our previous study, which showed that itraconazole was ineffective against *F. pedrosoi* biofilm-forming cells (initial inoculum of 10^4^ conidial cells), presenting bMIC value higher (4200-fold) than planktonic cells [17]. In fact, studies have shown that biofilm-forming cells are at least 1000-fold more resistant to antimicrobial drugs than planktonic cells [63]. Interestingly, *F. pedrosoi* biofilm, even during its formation and maturation, were less resistant to both silver(I)-phen complexes than to antifungal drugs, as we observed for itraconazole (data not shown) and published previously [17]. 

**Figure 2:**
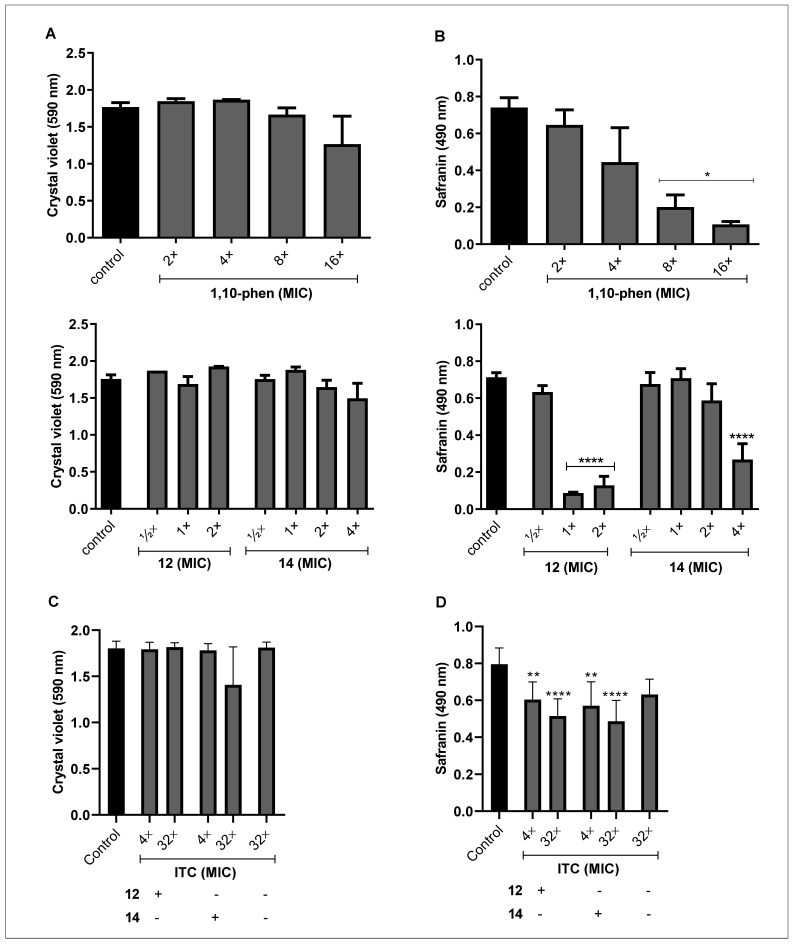
Effect of phen and its silver complexes on *F. pedrosoi* biomass and extracellular matrix during biofilm formation. (**A**,**B**) Conidia were added to 96-well microplates containing RPMI medium and non-cytotoxic concentrations of each compound. (**C**,**D**) Conidia were treated with a combination of non-inhibitory concentrations of itraconazole and the complexes **12** (½ MIC) and **14** (2×MIC). The systems were incubated for 72 h at 37 °C and then biofilm biomass (**A**,**C**) and extracellular matrix (**B**,**D**) were quantified by incorporation of crystal violet and safranin, respectively. Systems containing only non-treated conidia were also prepared (control). * *p* < 0.05, ** *p* < 0.01, **** *p* < 0.0001.

The scarce antifungal activity against biofilm-forming cells can be attributed to several structural components responsible for biofilm development and its maintenance, including polysaccharides, (glyco)proteins, (glyco)lipids and extracellular DNA, essential constituents of extracellular matrix [63,64]. Thus, we evaluated the effect of phen and its silver complexes on *F. pedrosoi* biomass and extracellular matrix. The results revealed that the incorporation of crystal violet was not affected by the incubation with non-inhibitory concentrations, indicating that during the biofilm formation, no alteration in the amount of biomass was detected (Figure 2A). On the contrary, silver(I)-phendione complex (**10**) at a non-cytotoxic concentration was able to disrupt the biofilm biomass of *P. verrucosa* [20]. However, the safranin staining suffered a significant reduction (Figure 2B). For instance, the biofilm of *F. pedrosoi* treated with phen (16×MIC value), the complexes **12** (2×MIC) and **14** (4×MIC) had its extracellular matrix decreased around 85%, 80% and 60%, respectively (Figure 2B). Conversely, we showed that phen and its silver complexes, as well as itraconazole, were not able to disturb either biomass or extracellular matrix of the mature biofilm produced by *F. pedrosoi* under the conditions tested (data not shown). 

The reduction on biofilm viability and extracellular matrix without significantly affecting the biomass, using these standard protocols to measure biofilm parameters, can be associated with many possible explanations. For example, silver complexes modulate the differentiation process, which culminates in arresting the conidia into mycelia transition. In addition, conidia and mycelia present different metabolism and viability, which is also dependent on the time in culture and cell cycle phase. Moreover, the production of cell-surface and extracellularly released molecules by both fungal morphotypes are distinct. This is well-known based on available literature, and, of course, the treatment with the test compounds can modulate the production of cell-surface and extracellular molecules which interfere with crystal violet and safranin binding properties. In this context, it is relevant to highlight that the main difference between crystal violet and safranin protocols is that for the first one, the fungal cells are fixed in methanol before staining. In crystal violet assay, methanol fixation is used to avoid detachment of the biofilm during the staining and rinsing steps. Perhaps after compound treatment, this fixation had reduced the extracting of stains from the biofilm compared to safranin staining that does not have a methanol fixation step. This can explain, at least in part, the difference of our results observed in safranin and crystal violet assays. Thus, the results are multimodal events and required more detailed experiments in order to, for example, measure the number of conidia and filamentous form inside the biofilm, the ability of these cells to produce biofilm-related molecules, and the ability of these test compounds to penetrate the biofilm structure and in both distinct fungal morphotypes.

The resistance attributed to microbial biofilm is multifactorial and may be associated with, for instance, (i) the difficulty for the drug to penetrate the cells; (ii) overcoming the barrier generated by the extracellular matrix; (iii) the genetic adaptation and (iv) the differences in the metabolic activity [65]. In order to minimize antimicrobial resistance, some studies have proposed the use of metal complexes in combination with classical antifungal drugs [21,66,67]. In this context, we also investigated the interaction of silver(I)-phen complexes and itraconazole, using non-inhibitory concentrations. Our data showed that the combination of the complex **14** (4×MIC [2.4 µM]) and itraconazole (32×MIC [12.5 µM]) was able to reduce about 70% of the *F. pedrosoi* biofilm viability (Figure 1C). The biofilm biomass was not significantly affected (Figure 2C), but the complex **14** at 2×MIC combined with different concentrations of itraconazole, 4×MIC (1.5 µM) and 32×MIC, disarticulated the extracellular matrix of *F. pedrosoi* around 30 and 40%, respectively (Figure 2D). While complex **12** (½×MIC [0.6 µM]) associated with itraconazole at 4×MIC and 32×MIC affected only the safranin incorporation around 25% and 35%, respectively (Figure 2D). 

The good performance of complex **14** was described previously for having inhibited the growth of different isolates of *Pseudomonas aeruginosa* producing biofilm, alone or in combination with gentamicin, an antibacterial agent [61]. Moreover, the interactions between silver(I) complexes with antimicrobial agents were able to inhibit the growth of fungal planktonic cells. Eshwika et al. [66] showed that *C. albicans* treated with silver(I)-phendione (complex **10**) combined with miconazole and amphotericin B had its viability more affected than when it was treated with each of them alone. In addition, Granato et al. [21] reported that the same complex combined with amphotericin B reduced 2-fold the MIC values found for each compound. It is well established that a combined therapy may increase the effect of drugs or act synergistically, inhibiting the planktonic cells growth, and even the biofilm formation or disarticulation. Thus, such an approach could minimize the resistance to drugs and their toxicity, making them more appropriate for clinical applications [68].

### 3.3. Effect of Silver Complexes on F. pedrosoi Differentiation

**Figure 3:**
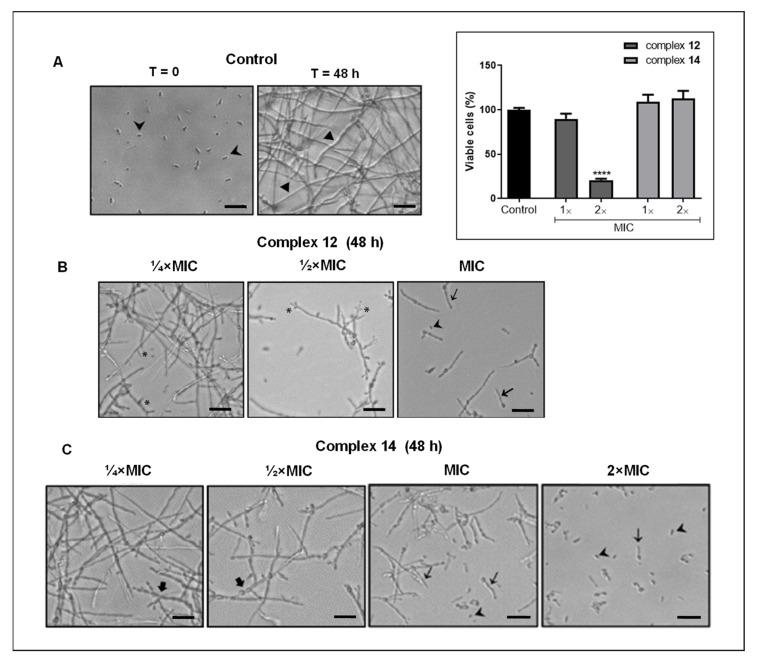
Effect of silver complexes on *F. pedrosoi* differentiation. (**A**) Control systems, conidia (⮞) in RPMI medium before (Time zero, T = 0) and after 48 h of incubation (T = 48 h), when filamentous form (►) was produced; (**B**) Conidia treated with ¼ and ½ MIC of complex **12** had the ability to branch (*) and form hyphae (►) inhibited after 48 h. In the MIC concentration, germ tubes (→) and conidia (⮞) were especially observed; and (**C**) Complex **14** (¼ and ½ MIC) inhibited hyphae formation, but moniliform cells (🡆) were found. While in the MIC and 2×MIC values, germ tubes (→) and conidia (⮞) were the most often detected. Bar: 10 µM. (**Inset**): The graph shows the viability of conidia after treatment with complexes using XTT assay [52]. **** *p* < 0.0001.

The effective inhibition of *F. pedrosoi* biofilm formation by silver complexes motivated us to analyze their capability to affect hyphae formation. Indeed, studies have revealed that filamentation is one of the first steps involved with biofilm production and it directly impacts the fungal pathogenicity [69,70]. We showed that both complexes (**12** and **14**), in the non-inhibitory concentrations, as determined using XTT assay (Figure 3, inset), were able to negatively modulate the transition of conidia into filamentous form in a typically dose-dependent manner. After 48 h, as expected, untreated conidia were fully transformed into mycelia (Figure 3A). In contrast, conidia cells treated with complex **12** at ¼×MIC and ½×MIC had the ability to branch and produce true hyphae inhibited (Figure 3B). Additionally, MIC concentration highly inhibited the fungal filamentation, thus, conidia and germ-tubes in particular were observed (Figure 3B). Conidia treated with complex **14** (¼×MIC and ½×MIC) were not able to form true hyphae, but moniliform hyphae was detected (Figure 3C). The fungal morphological transition was effectively inhibited by MIC and 2×MIC values of complex **14**, and germ-tubes and conidia were observed at the latter (Figure 3C). Likewise, we showed that another silver complex (**10**) containing phendione inhibited the *P. verrucosa* morphological transition [19]. Thus, our data corroborate that metal complex derived from phen are effective at disturbing the CBM fungi cell differentiation, an essential virulence attribute for the establishment of fungal infections [71,72].

### 3.4. Effect of Silver Complexes on Enzymes Produced by F. pedrosoi

It is well known that many attributes favor the fungal virulence, including the hydrolytic enzyme activities [22,73,74]. We previously showed that *F. pedrosoi* is capable of producing hydrolytic enzymes such as metalopeptidase, aspartic peptidase, ectophosphatase, phospolipase and esterase [9,10,11,13,14,15,16]. Considering that metal-based drugs can inhibit enzymes by mimicking substrates and metabolites [42], we assessed the action of silver(I)-phen complexes on enzymatic activities produced by *F. pedrosoi*. The results showed that metallopeptidase activity of *F. pedrosoi* was significantly affected by the treatment with both silver(I)-phen complexes, in which the complex **12** was more effective, inhibiting approximately 60% of the substrate catalysis, while complex **14** inhibited 40% (Table 4). It is known that metal chelating complexes may disturb the function of metallo-type enzymes [25,38]. Corroborating our current findings, phen and silver(I)-phendione (complex **10**) also inhibited the metallopeptidase activity of *P. verrucosa* [19]. The inhibition of metallopeptidases by phen is mainly due to its ability to chelate Zn^2+^ ions, crucial for its catalytic activities [11,25,75]. We previously showed that phen affected the proliferation and cell differentiation process of *F. pedrosoi*, suggesting that metallopeptidase may be involved in its biology and virulence [11,75]. Interestingly, the inhibition capacity of complexes **12** and **14** was solely higher than the capacity of phen. Indeed, both complexes inhibited the metallopeptidase activity secreted by *F. pedrosoi* by 50% in average, while the same percentage was only reached with 20-fold of 1 mM phen as we previously detected in Palmeira et al. [11]. 

Regarding *F. pedrosoi* aspartic peptidase, both silver complexes were also able to inhibit its activity at concentration of 50 µM. As shown for metallopeptidase, complex **12** was more effective, reducing around 44% compared to 35% of complex **14** (Table 4). The specificity of enzymatic hydrolysis was confirmed with the high blockage of activity (~90%) by pepstatin A (10 μM), a comercial aspartic peptidase inhibitor, as we previously showed in Palmeira et al. [15]. Kellett et al. [76] discussed the ability of some metallic compounds (metallocarborane, polyoxometalate and copper) acting as protease inhibitors, and their potential use as alternative drugs to organic protease inhibitors currently employed against protease-associated diseases. This review showed that metal-based agents have proteolytic enzymes as targets, are able to bind them and inhibit the wild-type HIV aspartic protease as well as parasitic proteases. We previously showed that the aspartic-type peptidase produced by *F. pedrosoi* conidia may be involved with crucial events, such as cellular growth and differentiation as well as interaction with host cells [15]. It is still unknown how the complexes can affect the peptidases produced by *F. pedrosoi*, but their inactivity can block signaling events and metabolic pathways, as a result of the inhibition of some fundamental biological processes for microbial cells [15,22]. 

In contrast, *F. pedrosoi* ectophosphatase activity, confirmed by high inhibition (90%) after treatment with sodium orthovanadate, a classical acid phosphatase inhibitor (data not shown), as previously described by Kneipp et al. [10] was not sensitive to complexes **12** and **14,** when the non-inhibitory concentrations were assayed (Table 4). In order to exclude the presence of the disrupted cells in the reaction after complexes treatment, the fungal viability was monitored using XTT colorimetric assay. Cells remained viable up to the MIC value of complex **12** and 2×MIC value of complex **14.** For this reason, this set of experiments cannot be carried out with the tested complexes in high concentrations (50 µM), as we did for the extracellular metallopeptidase assay using the culture supernatant. Then, our experiments do not rule out the possibility that the phosphatase activity may be affected by metal complexes in vivo while in contact with host cells. It is well known that enzymes involved in the phosphorylation control play key roles in fungal biology and pathogenesis [74]. We previously showed that *F. pedrosoi* surface phosphatase is involved in the adhesion to host cells, and may contribute to the early mechanisms required for CBM establishment [10]. The study of enzymatic inhibition mediated by metal compounds has significantly increased as a consequence of the development of these compounds’ applications in medicine [42]. Previous studies revealed that complexes containing metals, such as vanadium, copper and gold, for instance, can act as potent inhibitors of human-cell protein phosphatases [42,77,78]. 

Considering the *F. pedrosoi* phospholipase and esterase enzymes, the treatment with silver(I)-phen complexes in non-cytotoxic levels, as determined by XTT colorimetric assay, was not either able to affect significantly their activities up to the highest complexes concentrations (**12**, MIC and **14**, 2×MIC, Table 4). The same outcome was observed when these highest concentrations were tested under the post-treatment conditions (data not shown). These results contrast with other studies that demonstrated metallic compounds of platinum and ruthenium were able to inhibit animal cells phospholipase activity [79,80]. Likewise, compounds containing metals, such as iron, cobalt, nickel or copper(II), negatively modulated the esterase activity of human erythrocytes [81].

As mentioned, intact and viable cells were used for the ectophosphatase, phospholipase and esterase assays. Thus, it was not possible to test higher concentrations of the complexes, as performed for metallo- and aspartic peptidase experiments. Such conditions, in part, may explain the lack of activity of these complexes against *F. pedrosoi* ectophosphatase and lipases.

### 3.5. Effect of Silver Complexes on the Melanin Production by F. pedrosoi 

**Figure 4:**
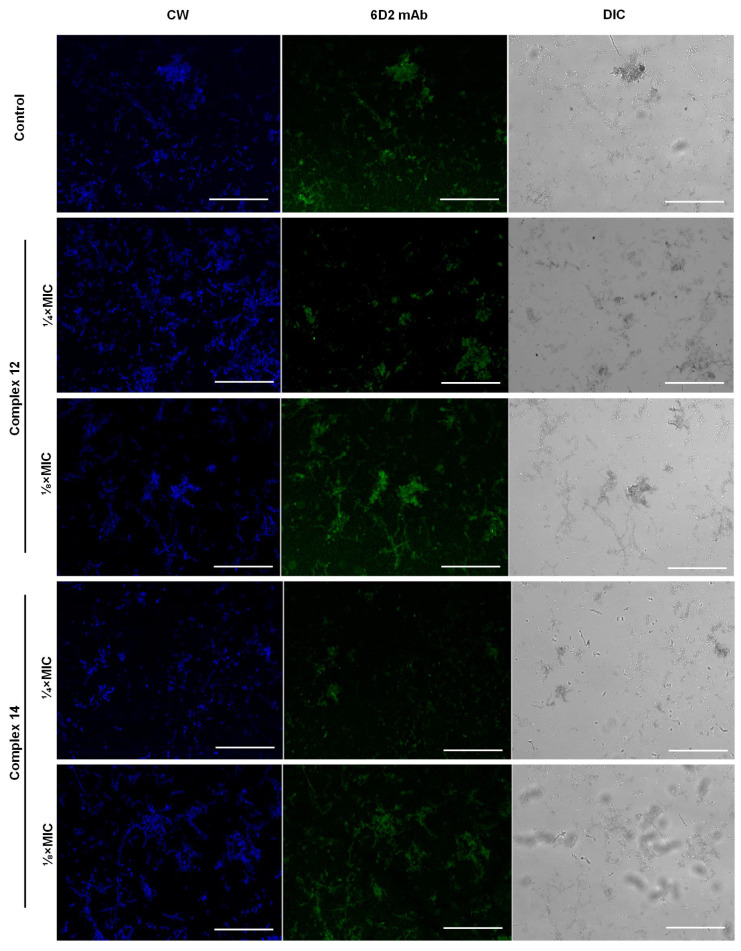
Effect of silver-phen complexes on *F. pedrosoi* melanin production. Fluorescence microscopy of fungal cells grown in the absence (control) and in the presence of silver complexes at different concentrations for 5 days at 26 °C, and then labeled with 6D2 mAb followed by incubation with anti-mouse IgM-Alexa 488 and calcofluor white (CW), as detailed in Material and Methods. Images were obtained using differential interference contrast (DIC) and fluorescence microscopy. Scale Bar: 50 µM. Note that all the cells treated with non-inhibitory concentration of ¼×MIC of both silver complexes were stained with calcofluor; however, most of them were not labeled with 6D2 mAb, showing that these cells had their melanin production inhibited.

Melanin is an important virulence factor for dematiaceous fungi, such as *F. pedrosoi*. The ability to produce melanin is related to the escape of fungal cells from phagocytosis, the resistance to oxidative stress and antifungal agents, among others [82]. Using fluorescence microscopy, we showed that 6D2 mAb originally produced against melanin derived from *Cryptococcus neoformans* also label *F. pedrosoi* cells, as previously reported for other filamentous fungi, such as *Sporothrix* spp. and *Paracoccidioides* spp. [57,83]. *F. pedrosoi* cells stained with calcofluor, which binds to chitin in the fungal cell walls and highlights its morphotypes, had their reactivity to 6D2 mAb reduced, indicating that melanin biosynthesis was affected, when they were treated with non-inhibitory concentration of ¼×MIC of both silver(I)-phen complexes (Figure 4). This finding is in contrast with Rossi et al. [83] that showed, using fluorescence microscopy and 6D2 mAb, that treatment of *Paracoccidioides* spp. with miltefosine, an analogue of alkylphospholipids at subinhibitory concentrations, was able to induce fungal melanization. Heidrich et al. [84], using CLSI, showed that the inhibition of *F. pedrosoi* DHN-melanin by TCZ increased the susceptibility to itraconazole, posaconazole and terbinafine. Although Coelho et al. [85] have shown a distinct *F. pedrosoi* susceptibility profile to antifungal drugs concerning DHN-melanin inhibition, they also reported that *Fonsecaea* spp. treated with TCZ had their MIC values of amphotericin B drastically reduced. Therefore, these bioactive drugs capable of inhibiting melanin, one of the most important virulence factors of black fungi, have great applicability to alternative approaches to treat CBM, especially when combined with classical antifungal agents [84].

### 3.6. Effect of Silver Complexes on the Production of ROS in F. pedrosoi 

**Figure 5:**
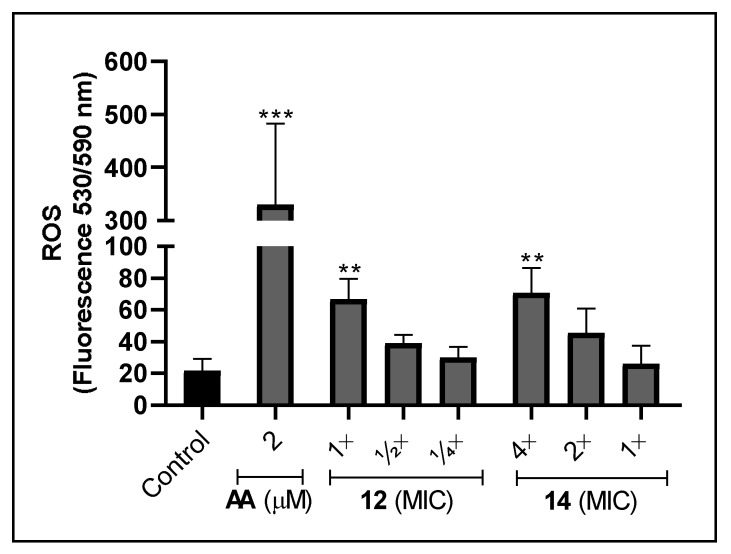
Conidia were incubated for 24 h at 26 °C in RPMI medium supplemented with distinct non-inhibitory concentrations of complexes **12** and **14.** The resorufin fluorescence was determined using Amplex™ Red Hydrogen Peroxide/Peroxidase Assay Kit and detected after reading in the fluorimeter. Antimycin A (AA) was used as positive control. ** *p* < 0.01, *** *p* < 0.001.

It has been shown that some metal-based drugs provoke microbial cell death by inducing ROS generation, such as superoxide anion and hydrogen peroxide [5,86]. Interestingly, ROS may react with transition metal and produce hydroxyl radical by Fenton reaction. As a consequence, they can cause cell death by binding and oxidizing essential molecules, such as proteins, lipids and DNA [5,87]. Thus, the maintenance of intracellular ROS balance is crucial for cellular growth and survival [88,89]. Bearing this in mind, the production of ROS after treatment with the silver(I)-phen complexes was evaluated in order to elucidate the possible mechanisms involved with anti-*F. pedrosoi* activity. Compared to non-treated cells, both complexes at the non-inhibitory concentrations (**12** at MIC [1.2 µM]) and **14** at 4×MIC [2.5 µM]) were able to induce around 3-fold the ROS production, after 24 h of incubation (Figure 5). Cells treated for 2 h with both complexes did not affect ROS rates (data not shown), while sublethal doses of antimycin, used as positive control, highly increased it (Figure 5). A previous study with *C. albicans* showed that the cooper(II)-malonate-phen complex generated oxidative stress, which was detected by increasing levels of lipid peroxidation and decreasing the ratio of reduced and oxidized glutathione [32]. Indeed, the mode of action of phen and its metal complexes have been associated with the mitochondrial function, inhibition of cytochrome biosynthesis and reduction of cellular respiration [30,32,33,37]. Additionally, it has already been described that silver perchlorate salt (AgClO_4_), was capable of inducing oxidative stress in fungal cells. In this study, Rowan et al. [90] demonstrated that the exposure of *C. albicans* to the AgClO_4_ resulted in the activation of oxidative stress by two pathways: the high osmolarity protein (Hog) and the activation protein (Cap). Thus, our data suggest that oxidative stress may be involved with the fungicidal effect generated by both complexes against *F. pedrosoi*.

## 4. Conclusions

Metal-phen complexes are emerging as a promising antifungal therapeutic approach, since they have different modes of action from the classical organic antifungal drugs, easy synthesis and low cost. Herein, the outcome revealed that several metal complexes derived from phen were able to affect the proliferation of species belonging to *Fonsecaea* genus. Among them, silver(I)-phen complexes were the most effective in inhibiting *F. pedrosoi* growth, the main etiological CBM agent. They also affected distinct potential virulence attributes of *F. pedrosoi*, such as its filamentation, metallo- and aspartic peptidases as well as melanin and biofilm production. Furthermore, *F. pedrosoi* conidia treated with silver(I)-phen complexes had its ROS production stimulated, suggesting that oxidative processes may be also associated with anti-*F. pedrosoi* action. Overall, the data corroborated that metal-phen complexes have the potential to be used as an alternative therapy to also combat CBM infections.

## Figures and Tables

**Table 1:** Metal-based complexes tested in the present study.

Complexes	Synthesis References
**Phthalic acid (phH_2_)**	
(**1**) [Mn(ph)(phen)(H_2_O)_2_]	Devereux et al., 2000 [43]
(**2**) [Cu(ph)(phen)(H_2_O)_2_]	Kellett et al., 2012 [44]
(**3**) [Cu(ph)(phen)_2_].3H_2_O.2EtOH *	Kellett et al., 2011 [45]
**Isophthalic acid (isophH_2_)**	
(**4**) [Mn_2_(isoph)_2_(phen)_3_].4H_2_O	Devereux et al., 2000 [43]
(**5**) [Cu(isoph)(phen)_2_].6H_2_O.EtOH	Kellett et al., 2011 [45]
**Terephthalic acid (terephH_2_)**	
(**6**) [Mn(tereph)(phen)_2_].5H_2_O	Salvadores 2000 [46]
(**7**) [{Cu(phen)_2_}_2_(terph)](terph).13.5H_2_O.2EtOH	Kellett et al., 2011 [45]
**Octanedioic acid (odaH_2_)**	
(**8**) [Mn_2_(oda)(phen)_4_(H_2_O)_2_][Mn_2_(oda)(phen)_4_(oda)_2_].4H_2_O	Casey et al., 1994 [47]
(**9**) [Cu_2_(oda)(phen)_4_](ClO_4_)_2_.2.76H_2_O.EtOH	Devereux et al., 1999 [48]
**Perchlorate salt (ClO_4_)**	
(**10**) [Ag(phendione)_2_]ClO_4_	McCann et al., 2004 [34]
(**11**) [Cu(phendione)_3_](ClO_4_)_2_.4H_2_O	McCann et al., 2004 [34]
(**12**) [Ag(phen)_2_]ClO_4_	McCann et al., 2004 [34]
**3,6,9-trioxaundecanedioate (3,6,9-tddaH_2_)**	
(**13**) {[Cu(3,6,9-tdda)(phen)_2_].3H_2_O.EtOH}n	Gandra et al., 2017 [36]
(**14**) [Ag_2_(3,6,9-tdda)(phen)_4_].EtOH	Gandra et al., 2017 [36]

* EtOH: Ethanol.

**Table 2:** Effect of metal complexes on planktonic growth of *Fonsecaea* spp.

Complexes	*F. monophora*	*F. nubica*	*F. pedrosoi*	
	MIC µM (mg/L)	MIC µM (mg/L)	MIC µM (mg/L)	MFC µM (mg/L)
**1**	25.0 (10.9)	25.0 (10.9)	6.2 (2.7)	>100.0 (>43.6)
**2**	100.0 (44.4)	>100.0 (>44.4)	>100.0 (>44.4)	>100.0 (>44.4)
**3**	100.0 (73.5)	50.0 (36.8)	50.0 (36.8)	>100.0 (>73.5)
**4**	ND	12.5 (13.2)	12.5 (13.2)	>100.0 (>105.6)
**5**	ND	ND	25.0 (18.6)	>74.5 (>100.0)
**6**	12.5 (8.5)	12.5 (8.5)	6.2 (4.2)	>100.0 (>67.1)
**7**	100.0 (146.0)	50.0 (73.0)	100.0 (146.0)	>100.0 (>146.0)
**8**	ND	ND	6.2 (15.5)	>100.0 (>248.3)
**9**	ND	ND	6.2 (8.2)	>100.0 (>131.6)
**10**	6.2 (3.9)	12.5 (7.9)	6.2 (3.9)	ND
**11**	6.2 (6.0)	3.1 (3.0)	3.1 (3.0)	3.1 (3.0) *
**12**	0.6 (0.3)	0.6 (0.3)	1.2 (0.7)	5.0 (2.8) *
**13**	6.2 (4.6)	3.1 (2.3)	3.1 (2.3)	>100.0 (>74.5)
**14**	1.2 (1.4)	2.5 (3.0)	0.6 (0.7)	2.5 (3.0) *

To determine the minimum inhibitory concentration (MIC_100_), the broth microdilution method was used as described in the document M38-A2 [49], with some modifications. The minimum fungicidal concentration (MFC) was calculated and the fungicidal effect (*) defined when MFC was ≤ 4 ×MIC value [51]. >100 μM means that, up to this concentration, 100% inhibition of fungal growth was not observed. Itraconazole, used as a reference antifungal drug, showed MIC of 0.39 μM (0.27 mg/L). DMSO used to dissolve itraconazole did not affect fungal growth. ND: Not determined.

**Table 3:** Effect of phen, phendione and different metal salts on the conidia *F. pedrosoi* viability.

Compounds	MIC µM (mg/L)	MFC µM (mg/L)
Phen	3.1 (0.6)	>100.0 (>19.3)
Phendione	3.1 (0.7)	>100.0 (>22.6)
Silver perchlorate	6.2 (1.9)	25.0 (7.7) *
Copper perchlorate	>100.0 (>37.0)	>100.0 (>37.0)
Silver nitrate	6.2 (1.1)	12.5 (2.2) *
Copper sulfate	>100.0 (>16.0)	>100.0 (>16.0)
Manganese chloride	>100.0 (>12.6)	>100.0 (>12.6)

To define the minimum inhibitory concentration (MIC_100_), the broth microdilution method was used as described in Material and Methods. Itraconazole used as a reference antifungal drug showed MIC of 0.39 μM (0.27 mg/L). The minimum fungicidal concentration (MFC) values were determined and the fungicidal effect (*) considered when MFC was ≤ 4×MIC value [51]. Concentrations >100 μM means that the fungal growth was not 100% inhibited up to this concentration. DMSO added to dissolve phendione and itraconazole did not affect fungal growth.

**Table 4:** Effect of silver complexes on *F. pedrosoi* enzymatic activities.

Complexes		Enzyme Activities						
	Aspartic Peptidase (%)	Metallo Peptidase (%)	Ectophosphatase (%)		Phospholipase Pz Value		Esterase Pz Value	
	50 µM	50 µM	2×MIC	MIC	2×MIC	MIC	2×MIC	MIC
**12**	56.0 ± 6.2 *	38.9 ± 5.5 *	ND	114.4 ± 8.0	ND	0.70 ± 0.03	ND	0.65 ± 0.01
**14**	65.3 ± 3.8 *	59.8 ± 6.9 *	99.3 ± 6.3	105.0 ± 7.5	0.64 ± 0.03	0.69 ± 0.02	0.72 ± 0.005	0.69 ± 0.01

The enzymatic activities were determined as detailed in Material and Methods. The metallopeptidase (90.52 ng FITC × h^−1^ × mg^−1^ protein), aspartic peptidase (11.929 μM MCA× h^−1^ × mg^−1^ protein) and ectophosphatase activities (6.25 nmol *p*-NP × h^−1^ × 10^7^ cells) detected in control systems were taken as 100%, and the values of the treated systems were converted to a percentage of the control values. The control systems of esterase (0.72 ± 0.01) and phospholipase (0.67 ± 0.03) were assessed by Pz indexes after 7 and 14 days of cultivation, respectively. The concentrations of both complexes, which were lower than MIC, did not affect the enzymatic activities (data no shown). ND: Not determined, since this concentration of complex **12** affected *F. pedrosoi* growth. * *p* < 0.05.
